# Azo-dimethylaminopyridine-functionalized Ni(II)-porphyrin as a photoswitchable nucleophilic catalyst

**DOI:** 10.3762/bjoc.16.179

**Published:** 2020-08-31

**Authors:** Jannis Ludwig, Julian Helberg, Hendrik Zipse, Rainer Herges

**Affiliations:** 1 Otto Diels Institute of Organic Chemistry, University of Kiel, Otto-Hahn-Platz 3-4, Kiel D-24098, Germany,; 2 Department of Chemistry, Ludwig-Maximilians-Universitaet Muenchen, Butenandtstrasse 5–13, 81377 Muenchen, Germany

**Keywords:** catalysis, Ni(II)-porphyrins, nucleophilic catalyst, photoswitch, record player molecules, spin switch

## Abstract

We present the synthesis and the photochemical and catalytic switching properties of an azopyridine as a photoswitchable ligand, covalently attached to a Ni(II)-porphyrin. Upon irradiation with 530 nm (green light), the azopyridine switches to the *cis* configuration and coordinates with the Ni^2+^ ion. Light of 435 nm (violet) isomerizes the ligand back to the *trans* configuration, which decoordinates for steric reasons. This so-called record player design has been used previously to switch the spin state of Ni^2+^ between singlet and triplet. We now use the coordination/decoordination process to switch the catalytic activity of the dimethylaminopyridine (DMAP) unit. DMAP is a known catalyst in the nitroaldol (Henry) reaction. Upon coordination to the Ni^2+^ ion, the basicity of the pyridine lone pair is attenuated and hence the catalytic activity is reduced. Decoordination restores the catalytic activity. The rate constants in the two switching states differ by a factor of 2.2, and the catalytic switching is reversible.

## Introduction

Photoswitchable catalysis has been realized following several approaches using a variety of photochromic systems. Feringa et al. recently published a review including systems based on double bond isomerizations [1]. An earlier review from the same group summarized light and redox responsive catalytic systems including azobenzenes, diarylethenes, spiropyranes, and stilbenes [2].

Diarylethenes were reported in the context of photoswitchable catalysis as inhibitors of the Karstedt´s catalyst [3] and for p*K*a modulation in acid–base-controlled processes [4]. Molecular motors for stereodivergent anion binding catalysis [5], azopeptides for the acetylation of sugars [6], enzyme mimics [7], and the utilization of intermolecular cooperative effects [8] are further applications of photoswitchable catalysis.

Particularly interesting and close to our approach is an early work by Inoue et al. who achieved control of the transformation of CO_2_ and 1,2-epoxypropane to propylene carbonate using an aluminum porphyrin and a photoresponsive ligand. The catalytic activity of the metal porphyrin depended on the axial coordination of an azostilbene and coordination of the latter ligand was controlled by photoisomerization of the stilbene unit [9].

Hecht et al. reported on the photoswitching of the basicity of a piperidine nitrogen by reversible steric shielding of the nitrogen lone pair. The photoswitchable base was applied as a nucleophilic catalyst in the nitroaldol (Henry) reaction. Attached to a sterically demanding azobenzene unit, the lone pair was shielded in the *cis* configuration and therefore catalytically inactive. The basicity was restored by isomerization to the *trans* state leading to a rapid conversion to the β-nitroalcohol, the product of the Henry reaction [10–12].

**Figure 1:**
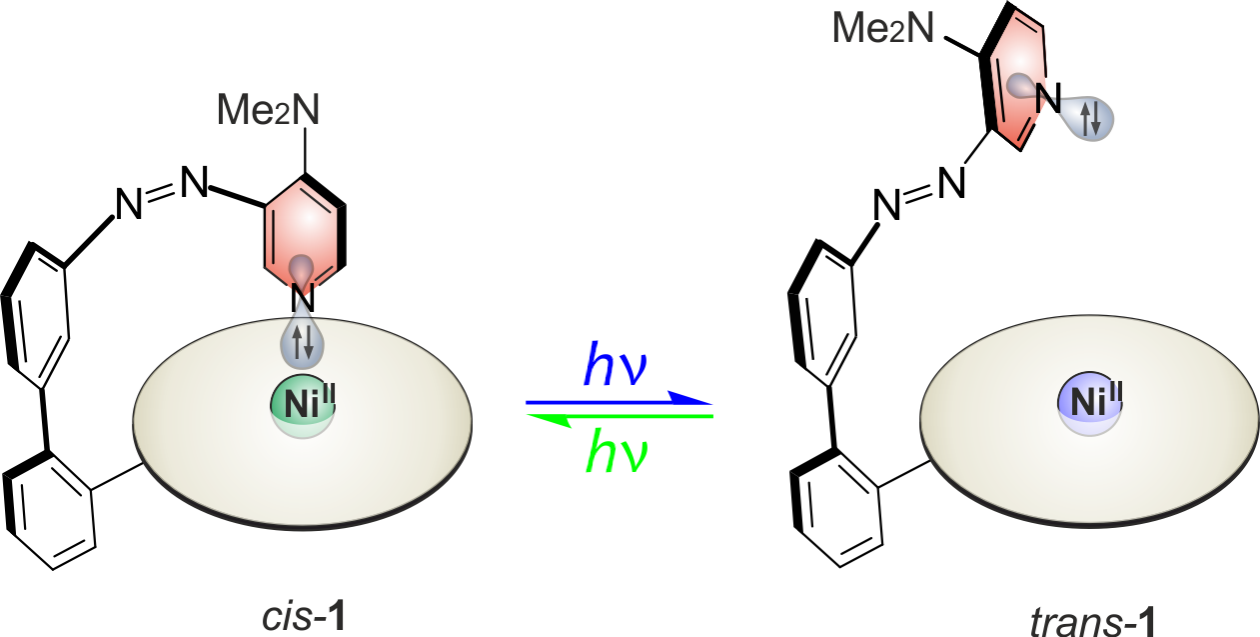
Basicity and nucleophilicity switching of the 4-(*N,N*-dimethylamino)pyridine “record player” molecule **1**.

We now present a photoswitchable catalyst whose basicity is controlled by coordination/decoordination to the Ni^2+^ ion in a Ni-porphyrin. So-called record player molecules, including a Ni(II)-porphyrin as the square planar base complex and azopyridines as photoswitchable axial ligands, were previously investigated for spin switching applications [13–26]. In the present study, the basicity change linked to the coordination/decoordination process is explored. If the azo substituent is in *cis* configuration, the lone pair of the pyridine unit coordinates to the central Ni^2+^ ion, reducing the nucleophilic power and the basicity of the pyridine (Figure 1). In the *trans* configuration intramolecular coordination is prohibited and nucleophilicity and basicity are restored. Reversible switching between the two states is achieved by irradiation with green (*trans*→*cis*) and violet (*cis*→*trans*) light.

The title compound of this study is 4-(*N,N*-dimethylamino)pyridine derivative **1** (Figure 1). The parent 4-(*N,N*-dimethylamino)pyridine (DMAP, **2**) is known as a nucleophilic catalyst in a number of reactions, for example the Baylis–Hillman reaction [27] and the Steglich esterification [28–29]. To achieve control of the catalytic activity of DMAP, dimethylamino record player **1** was investigated as a photoswitchable DMAP catalyst in the nitroaldol reaction of 4-nitrobenzaldehyde and nitroethane (Henry reaction) as a model reaction.

## Results and Discussion

### Synthesis

For the synthesis of 4-*N*,*N*-dimethylamino record player molecule **1**, a modular approach described by Heitmann et al. was chosen [30] since the alternative mixed aldehyde route failed for related porphyrin derivatives [17]. Ni-porphyrin precursor **3** and 4-(*N*,*N*-dimethylaminoazo)pyridine **4** were synthesized according to literature procedures [17,30].

**Scheme 1:**
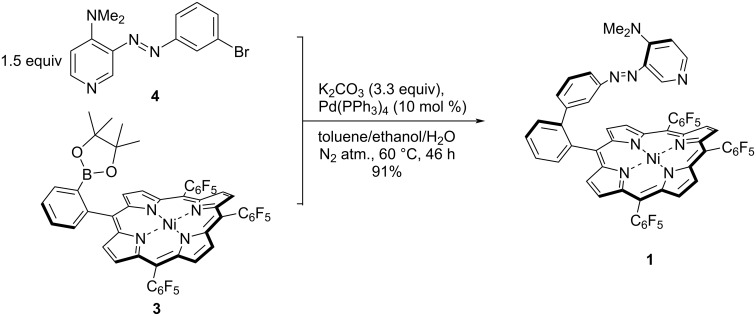
Synthesis of 4-*N*,*N*-dimethylamino record player molecule **1** by Suzuki reaction between Ni-porphyrin precursor **3** and bromo-substituted 4-(*N*,*N*-dimethylaminoazo)pyridine **4**.

Following the conditions for the Suzuki cross-coupling reaction published for a similar system [30], decomposition products and only 25% yield were obtained. Optimization by lowering the reaction temperature increased the yield to 91% (Scheme 1).

### Switching properties of porphyrin **1**

**Figure 2:**
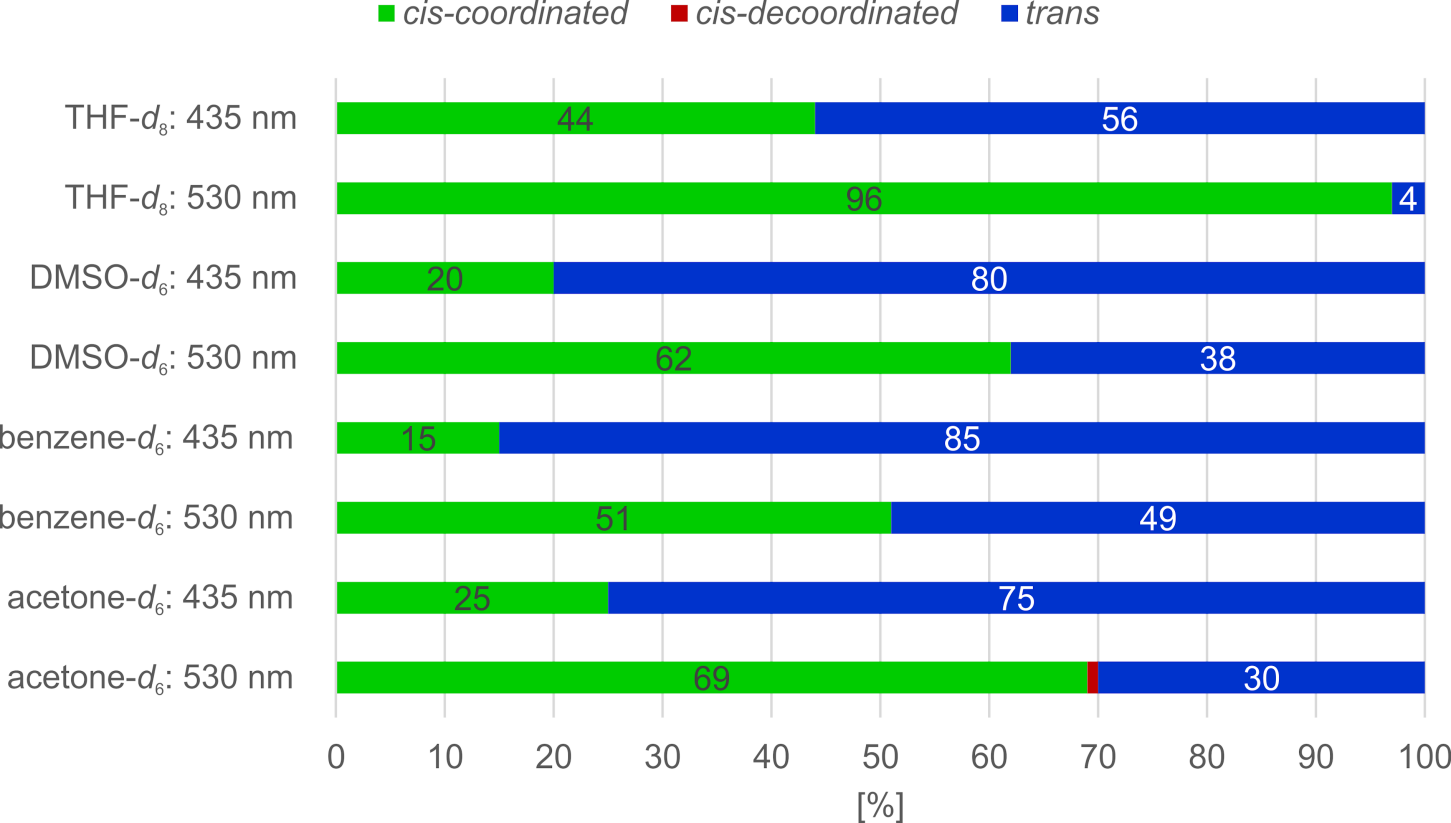
Composition of the different states of porphyrin **1** (1 mM) in the PSS at 530 nm and 435 nm, determined by ^1^H NMR spectroscopy (600 MHz; for details, see Supporting Information File 1).

**Figure 3:**
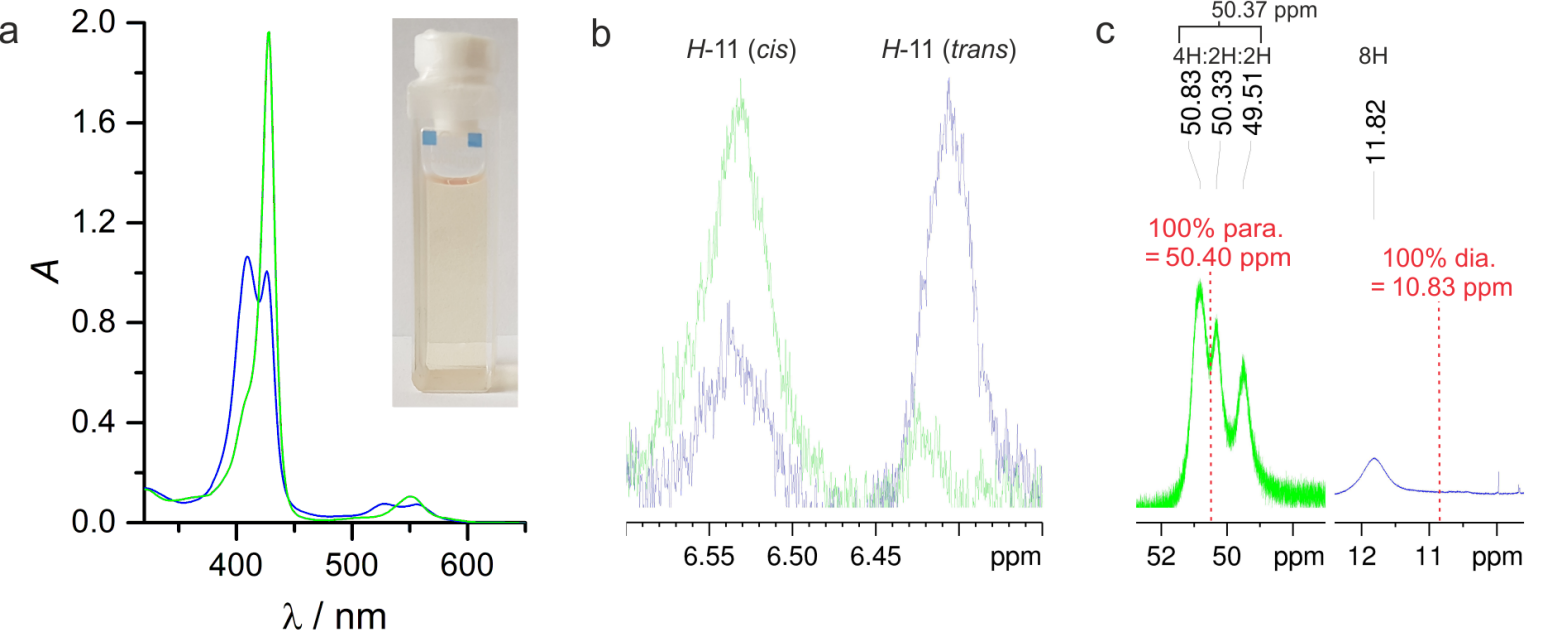
a) UV–vis cuvette with a solution of porphyrin **1** (13.1 µM in THF) and the corresponding UV–vis spectra at 25 °C in the PSS at 530 nm (green) and 435 nm (blue). b) Signals of proton H-11 (^1^H NMR, 600 MHz, THF-*d*_8_, 318 K), which are an indicator for the *cis–trans* isomerization of the azo unit of porphyrin **1**, in the PSS at 530 nm (green) and 435 nm (violet-blue). The signal at 6.54 ppm belongs to the *cis* species while the signal at 6.41 represents the *trans* isomer. c) ^1^H NMR signals (^1^H NMR, 600 MHz, THF-*d*_8_, 300 K) of the pyrrole protons in the PSS at 530 nm and 435 nm. Coordination/decoordination is fast on the NMR timescale and signal averages for the pyrrole protons are observed. 50.40 ppm corresponds to 100% coordinated (paramagnetic) Ni-porphyrin and 10.83 ppm is the chemical shift of the pure decoordinated (diamagnetic) system. The signals are broad because of paramagnetic line broadening of the coordinated species and fast ligand exchange.

Preliminary investigations on the photochemical switching properties of porphyrin **1** were performed to optimize the reaction conditions for the catalytic process. Among the different switching wavelengths investigated, 530 nm (*trans*→*cis*) and 435 nm (*cis*→*trans*) provided the largest conversion rates (Figure S1 in Supporting Information File 1). The influence of the solvent on the switching efficiency was evaluated by UV–vis (Figure S2, Supporting Information File 1) and NMR experiments (Figure 2). It is important to note that there are two ways to define the switching efficiency of record player molecules: 1. the *cis–trans* isomerization of the azopyridine unit, and 2. the coordination–decoordination process. Both switching processes are connected by the molecular design; however, they are not necessarily equally efficient because the *cis* isomer might not coordinate completely and there might be coordination in the *trans* state due to intermolecular coordination. On this account, isomerization and coordination were investigated separately by ^1^H NMR spectroscopy. The protons *meta* to the azo group in the azopyridine unit (H-11) are responsive to the configuration of the azo group (*cis* or *trans*) and the chemical shifts of the pyrrole protons of the porphyrin ring are strongly dependent on the axial coordination at the central Ni^2+^ ion (Figure 3, and for details see Tables S1 and S2 in Supporting Information File 1). The analysis of the NMR data revealed that 98.8–99.9% of the *cis* isomer is coordinated in all solvents, which is expectable because DMAP is a very strong ligand with respect to axial coordination to Ni-porphyrins [25]. According to ^1^H NMR studies, however, the *trans* isomer exhibits concentration-dependent coordination, which clearly indicates intermolecular coordination. Again, this is due to the very strong coordination power of the DMAP ligand. At a total concentration of 4 mM of the Ni-porphyrin, after irradiation with 435 nm (THF-*d*_8_, 25 °C) 46% of the molecules are in *cis* and 54% are in *trans* configuration. Whereas the *cis* isomers are almost completely coordinated, the *trans* porphyrins still exhibit 7% coordination, which is due to a fast intermolecular ligand exchange [14]. At a total porphyrin concentration of 40 mM, the intermolecular coordination of the *trans* species increases to 25%. Taking the incomplete photochemical conversion to the *trans* configuration into account (60%), only 45% of the Ni-porphyrin species should be catalytically active, if a solution of 40 mM total porphyrin concentration is applied.

The photostationary states after irradiation with 435 and 530 nm are strongly solvent dependent (Figure 2). THF turned out to provide the largest conversion rate (96%) to the coordinated (*cis*) state [31]. The back-reaction with 56% decoordination (*trans* isomer) is less efficient. At the other end of the scale, in acetone only 69% coordination (530 nm) and 75% decoordination (435 nm) is achieved. It should be noted that a high conversion to the inactive state (in photocatalysis as well as photopharmacology) is more important than achieving a high conversion to the active state because the latter problem can be compensated by increasing the concentration, whereas incomplete deactivation is a problem, which cannot be circumvented. Hence, THF was chosen as the solvent of choice for the catalysis experiments.

A decreasing switching efficiency of **1** at 530 nm from *trans* to *cis* was observed at higher concentrations (83% *cis*-**1** at 40 mM total porphyrin), whereas the switching to the *trans* state is almost unaffected (60%, Table S3 in Supporting Information File 1). The reduced switching efficiency from the *trans* to the *cis* isomer at higher concentrations can be explained by the intermolecular coordination, which stabilizes the *trans* isomer.

Long-term switching experiments were performed to investigate the fatigue resistance of **1**. In contrast to other record player molecules, which are stable up to 100 000 switching cycles, the switching efficiency slowly decreased over 100 switching cycles to ≈80% of the original value (see Supporting Information File 1, Figure S4). A thermal decomposition at 40 °C in THF was observed by a color change from red to green. Hence, the catalytic experiments were limited to 25 °C.

At last, the thermal relaxation time for the *cis→trans* isomerization in THF was determined by ^1^H NMR spectroscopy. Similar to previous record player systems a long half-life of the metastable *cis* isomer of about 505 d (25 °C, THF-*d*_8_) was determined (Figure S5 in Supporting Information File 1). Thus, the thermal relaxation of the coordinated *cis* state is insignificant for our catalytic studies.

### Experiments using porphyrin **1** as a catalyst

**Scheme 2:**
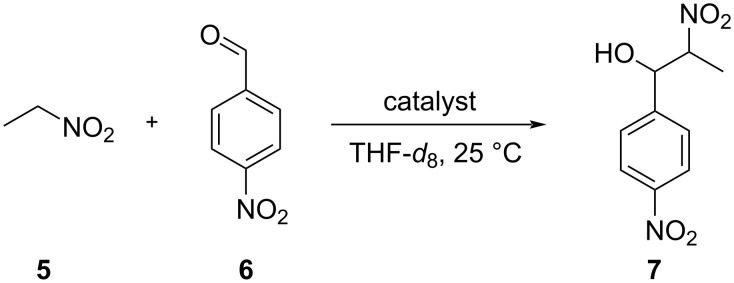
General scheme of the nitroaldol (Henry) reaction that was used to investigate photoswitchable catalysis. The following reaction conditions were applied: (0.4 M 4-nitrobenzaldehyde (**6**), 12 equivalents of nitroethane (**5**), and 4 mM or 40 mM of catalyst (see Scheme 3) at 25 °C in THF-*d*_8_) to yield **7**. For details see Tables S4 and S5 in Supporting Information File 1.

The Henry reaction (Scheme 2) was chosen as a model reaction to investigate the catalytic activity of porphyrin **1** in the two different switching states because the rate of the background reaction without catalyst is low, and the influence of the solvent on the reaction rate is small [10,32].

No catalytic effect for the N=N functional group of azobenzenes was previously observed in similar systems [10]. Ni-porphyrin Ni-TPPF_20_ **8** (without azopyridine substitution) does not catalyze the reaction either (Figure S6, Supporting Information File 1). Thus, it should be obvious that any catalytic effect, if detected, must be due to the DMAP unit.

**Scheme 3:**
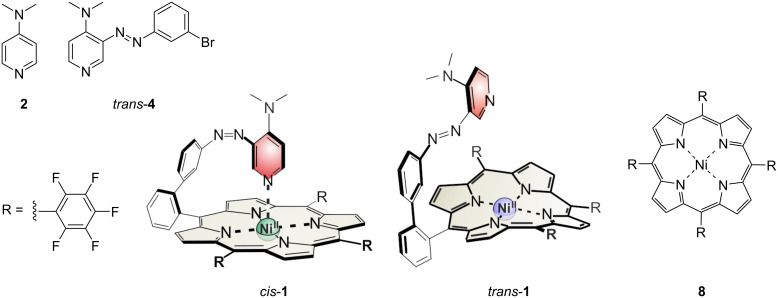
DMAP (**2**), azopyridine *trans-***4**, record player *trans*- and *cis-***1** and Ni-porphyrin **8** were used in kinetic experiments.

In our kinetic experiments, the following compounds were used as catalysts or reference compounds: record player **1** in the PSS at 530 nm (*cis*/*trans* ratio: 93:7 at 4 mM and 83:17 at 40 mM total concentration of **1**) and 435 nm (*cis*/*trans* ratio: 46:54 for 4 mM and 40:60 at 40 mM), DMAP **2**, azopyridine **4**, and Ni-porphyrin **8** (Scheme 3 and Figures S8 and S9, Supporting Information File 1).

**Figure 4:**
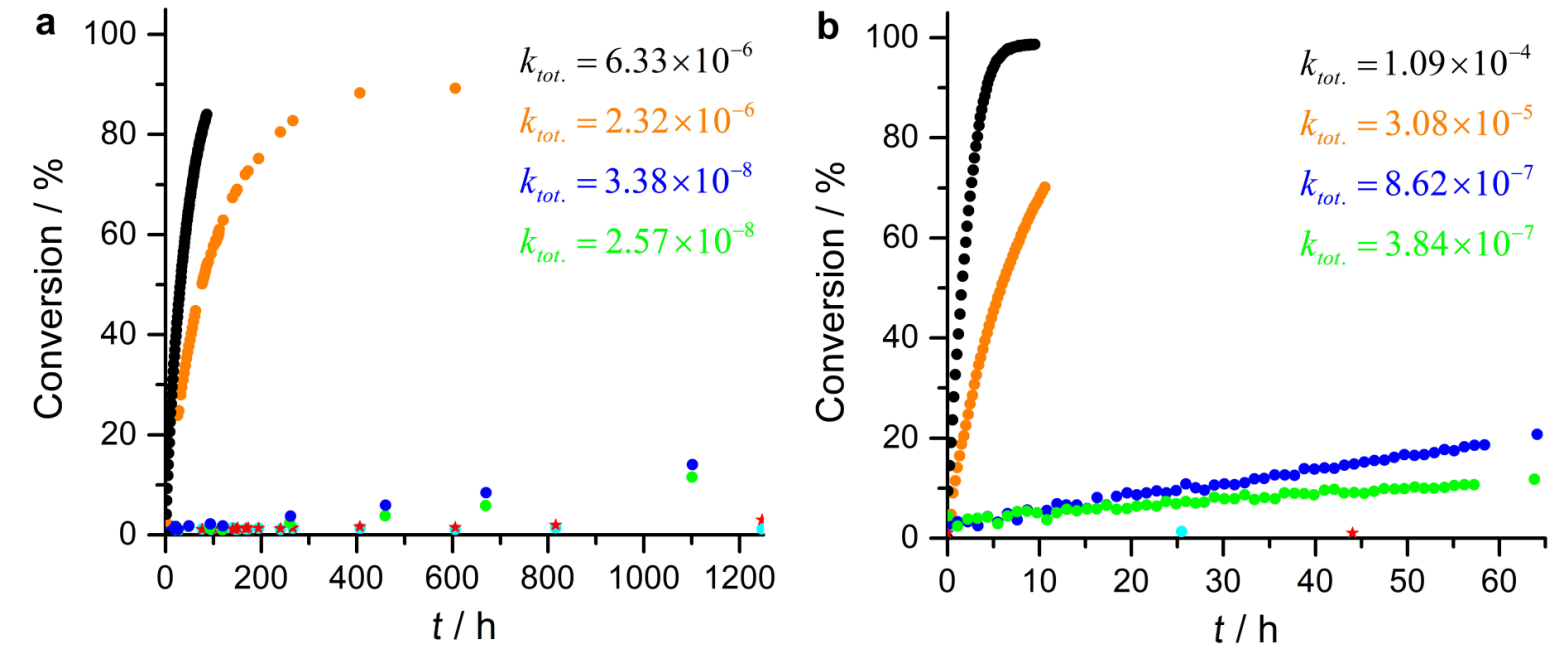
Conversion of 4-nitrobenzaldehyde (**6**) in the Henry reaction with nitroethane (**5**) as a function of time, using (a, left) 4 mM concentration of catalyst or reference compound (1 mol % compared to 4-nitrobenzaldehyde) or 40 mM catalyst (10 mol % compared to 4-nitrobenzaldehyde) (b, right). Rate constants (*k*_tot_) are given for DMAP (**2**) (black dots), azopyridine *trans-***4** (orange), photoswitchable porphyrin **1** at PSS_435nm_ (predominantly *trans-***1**, blue), and **1** at PSS_530nm_ (predominantly *cis-***1**, green). The background reaction was determined with porphyrin **8** (cyan dots) and for a sample without catalyst or reference compounds (red stars).

All catalysts and reference compounds were used in two concentrations 4 mM (1 mol % with respect to 4-nitrobenzaldehyde (**6**, Figure 4a) and 40 mM (10 mol %, Figure 4b). As stated above, intermolecular coordination of *trans*-**1** increases at higher concentrations (7% at 4 mM and 25% at 40 mM) and the *trans*→*cis* conversion drops from 93 to 83% at 40 mM concentration of **1**. So one can expect that the catalytic switching efficiency of **1** should drop at higher concentrations.

As expected, DMAP **2** exhibits the highest catalytic activity. The azo-substituted DMAP *trans*-**4** is slightly less effective because of the electron-withdrawing effect of the azo group. Surprisingly, however, the catalytic effect drops by a factor of 54 (4 mM pure *trans*-**1** vs *trans*-**4**) or by a factor of 18 (40 mM pure *trans*-**1** vs *trans*-**4**), if the azopyridine unit of *trans*-**4** is attached to the porphyrin core as realized in *trans*-**1**. This is unexpected because any electron-withdrawing effect of the electron poor porphyrin should be weak. There are eight bonds between the porphyrin β-position and the pyridine ring including one *meta*-phenyl connection attenuating the through-bond conjugation. A closer look at the kinetic data in Table 1 (sample I, II, and VII) might give an explanation for the surprisingly low catalytic activity of *trans*-**1**. While the background reaction of the Henry reaction is very slow but still measurable, the addition of Ni-porphyrin **8** inhibits this reaction completely (see also Supporting Information File 1, Figure S8). We propose that the actual nucleophile in the Henry reaction, the nitroethane anion intermediate, coordinates to the Ni-porphyrin, which slows down nucleophilic addition to the nitrobenzaldehyde. Hence, the switchable catalyst **1** contains both, a catalytic center (nitrogen lone pair) and an inhibitor (Ni^2+^ ion). If the nitrogen lone pair coordinates to the Ni^2+^ ion in the *cis* configuration of **1**, both reactive centers keep each other at bay. The isomerization to the *trans* configuration unleashes the catalytic as well as the inhibitory properties of **1**.

**Table 1:** Pseudo-first order rate constants (*k*) of the Henry reaction (Scheme 2) in the presence of different catalysts and reference compounds. Calculated rate constants are based on the assumption of complete isomerization and no intermolecular coordination. The concentration of the catalyst is given in mol % (in parentheses) with respect to the initial nitrobenzaldehyde (**6**) concentration.

sample	catalyst (mol %)	*cis/trans ratio*	*k* [s^−1^]

I	blank (0%)	–	3.32 × 10^−9^
II	Ni-TPPF_20 _**8** (1%)	–	–^a^
III	DMAP **2** (1%)	–	6.33 × 10^−6^
IV	*trans*-NMe_2_-azopyridine **4** (1%)	0:100	2.32 × 10^−6^
V	530 nm PSS: NMe_2_-record player **1** (1%)	93:7	2.57 × 10^−8^
VI	435 nm PSS: NMe_2_-record player **1** (1%)	46:54	3.38 × 10^−8^
calc.^b^	*cis*-NMe_2_-record player **1** (1%)	100:0	(2.45 × 10^−8^)^b^
calc.^b^	*trans*-NMe_2_-record player **1** (1%)	0:100	(4.30 × 10^−8^)^b^
VII	Ni-TPPF_20 _**8** (10%)	–	–^a^
VIII	DMAP **2** (10%)	–	1.09 × 10^−4^
IX	*trans*-NMe_2_-azopyridine **4** (10%)	0:100	3.08 × 10^−5^
X	530 nm PSS: NMe_2_-record player **1** (10%)	83:17	3.84 × 10^−7^
XI	435 nm PSS: NMe_2_-record player **1** (10%)	40:60	8.62 × 10^−7^
calc.^b^	*cis*-NMe_2_-record player **1** (10%)	100:0	(1.95 × 10^−7^)^b^
calc.^b^	*trans*-NMe_2_-record player **1** (10%)	0:100	(1.68 × 10^−6^)^b^

^a^Very slow, not measurable; ^b^assuming complete isomerization and no intermolecular coordination. For details see Supporting Information File 1, section IV.

Nevertheless, the reaction was faster after irradiation of the catalyst **1** (4 mM in THF-*d*_8_) with 435 nm (54% *trans*, 46% *cis* isomer) as compared to the catalyst **1** irradiated with 530 nm (7% *trans*, 93% *cis* isomer) by a factor of 1.3. At a concentration of **1** of 40 mM the photoswitchable catalyst at PSS_435nm_ (60% *trans*, 40% *cis*) is 2.25 times more efficient as the catalyst at PSS_530nm_ (17% *trans*, 83% *cis*) (Figure 4a and b). The theoretical reaction rate *k*_0_ of the nitroaldol reaction with 100% uncoordinated *trans*-**1** (40 mM) is 8.6 times higher than the reaction rate with pure *cis*-**1**. Thus, incomplete conversion and intermolecular coordination are further reasons for the relatively low switching efficiency.

## Conclusion

A photoswitchable base catalyst for the nitroaldol (Henry) reaction was synthesized and investigated. Principle of function is the control of the basicity of a DMAP derivative by light-induced coordination/decoordination of an azo-DMAP unit covalently bound to a Ni-porphyrin. Upon irradiation with 530 nm (green) light and 435 nm (violet) light, the nitroaldol reaction was accelerated/decelerated by a factor of 2.25. One of the factors reducing the catalytic activity and switching efficiency, obviously, was the deactivation of the nitroalkyl anion intermediate by coordination to the Ni-porphyrin. The DMAP-catalyzed acetylation of alcohols should not suffer from this drawback because the reactive intermediate (acylpyridinium cation) is positively charged and does not coordinate to the Ni^2+^ ion [33]. Another promising approach towards coordination-based, photoswitchable catalysts are photodissociable ligands. These ligands coordinate in their *trans* form and decoordinate in the *cis* configuration driven by steric hindrance [14,34–35].

## Supporting Information

File 1 General information, synthetic and photophysical procedures, and copies of NMR spectra.
