# Supplementary material for: Azo-dimethylaminopyridine-functionalized Ni(II)-porphyrin as a photoswitchable nucleophilic catalyst
Source: Beilstein J Org Chem. 2020 Aug 31;16:2119–26. doi: 10.3762/bjoc.16.179 (PMC7476594; doi:10.3762/bjoc.16.179)
Supplement: File 1 — General information, synthetic and photophysical procedures, and copies of NMR spectra. [file Beilstein_J_Org_Chem-16-2119-s001.pdf]

BEILSTEIN JOURNAL OF ORGANIC CHEMISTRY

Supporting Information

for

Azo-dimethylaminopyridine-functionalized Ni(II)-porphyrin as a photoswitchable nucleophilic catalyst

Jannis Ludwig, Julian Helberg, Hendrik Zipse and Rainer Herges

Beilstein J. Org. Chem. **2020,** *16,* 2119–2126. doi:10.3762/bjoc.16.179

General information, synthetic and photophysical procedures, and copies of NMR spectra

License and Terms: This is a supporting information file under the terms of the Creative Commons Attribution License (https://creativecommons.org/licenses/by/4.0). Please note that the reuse, redistribution and reproduction in particular requires that the authors and source are credited.
The license is subject to the *Beilstein Journal of Organic Chemistry* terms and conditions: (https://www.beilstein-journals.org/bjoc)

Table of contents

I Equipment

II Synthetic procedure

III Procedure for photophysical experiments

IV Catalysis: Experimental procedure

V NMR spectra

VI References

I: Equipment

Nuclear magnetic resonance (NMR) spectroscopy

The ^{1}H NMR measurements were carried out on a Bruker Avance 200, a Bruker AvanceNeo 500 or a Bruker Avance 600 spectrometer at 200 MHz, 500 MHz or 600 MHz in deuterated solvents. The solvent residual signals were used as internal reference (acetone-d_6: δ = 2.05 ppm, benzene-d_6: δ = 7.16 ppm, $CDCl_3$: δ = 7.26 ppm, CD_2Cl_2: δ = 5.32 ppm, DMSO-d_6: δ = 2.50 ppm, THF-d_8: δ = 1.72 ppm) [1]. ^{13}C NMR measurements were carried out on the named spectrometers at 126 MHz or 151 MHz and the solvent residual signals were used as internal reference (acetone-d_6: δ = 29.84 ppm, benzene-d_6: δ = 128.06 ppm, $CDCl_3$: δ = 77.16 ppm, CD_2Cl_2: δ = 53.84 ppm, DMSO-d_6: δ = 39.52 ppm, THF-d_8: δ = 25.31 ppm) [1]. Standard 2D-spectra (COSY, HSQC, HMBC) were used to assign the signals. ^{19}F NMR measurements were carried out on a Bruker AvanceNeo 500 spectrometer at 471 MHz. Samples containing deuterated trifluoroacetic acid (TFA-d_1) to make substances soluble or completely diamagnetic are especially labelled.

Mass spectrometry (MS)

High resolution electrospray ionization mass spectrometry (ESIHRMS) was carried out on a Thermo Fisher Scientific Q Exactive™ UHMR Hybrid Quadrupole-Orbitrap™ mass spectrometer. Matrix-assisted laser desorption/ionization time of flight mass spectrometry (MALDI-TOF-MS) was carried out on a Bruker-Daltonics Biflex III mass spectrometer using 4-chloro-α-cyanocinnamic acid (Cl-CCA) as matrix.

UV–vis spectroscopy

Ultraviolet visible (UV–vis) spectra were recorded on a Perkin Elmer Lambda 14 spectrometer with a Lauda Ecoline Staredition 003 E100 thermostat or a Perkin Elmer Lambda 650 spectrometer with a Quantum Northwest Inc. qpod 2e sample holder in quartz cuvettes with a path length of 10 mm.

Irradiation modules

Samples were irradiated with an irradiation module containing 12 Roithner VL-440-Emitter LEDs (435 nm) and 12 Nichia NS6G083 LEDs (530 nm) manufactured by Sahlmann Photochemical Solutions. During the irradiation process samples were cooled by an airstream if necessary cooled over liquid nitrogen.

Infrared (IR) spectroscopy

Fourier transform infrared spectroscopy (FTIR) was carried out on a Perkin Elmer Spectrum 100 spectrometer with A531-G Golden-Gate-Diamond attenuated total reflection (ATR) unit. The signal intensity is given as s = strong, m = medium, w = weak, br = broad.

Melting points

Melting points (mp) were measured using a Büchi Melting Point M 560 in one-sided open tubes and are given uncorrected in °C.

Chromatography

Silica gel (Merck, particle size 0.040–0.063 mm) was used for column chromatography. R_f values were determined by thin layer chromatography on Polygram® Sil G/UV_{254} (Macherey-Nagel, 0.2 mm particle size).

II: Synthetic procedures and NMR spectra

Synthesis of 5-(biphenylazo-*N,N*-dimethylaminopyridine)-10,15,20-tris(pentafluorophenyl)nickel(II)porphyrin (1)

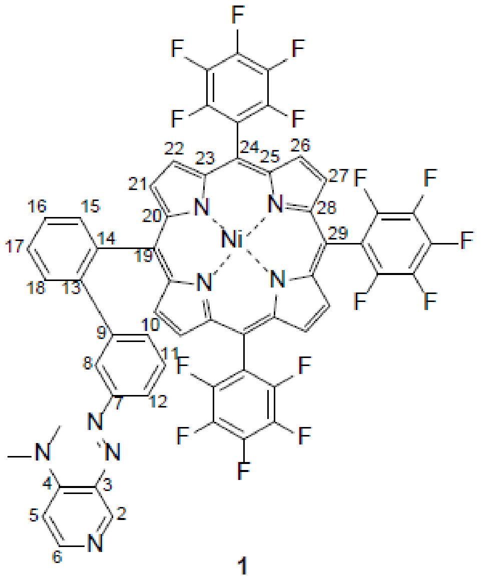

1

The solvent (30 mL toluene, 10.5 mL ethanol, 7 mL deionized water) was flushed with nitrogen for 10 min. Ni-Porphyrin precursor (200 mg, 187 µmol, **3**), 3-(3-bromophenylazo)-4-*N,N*-dimethylaminopyridine (85.8 mg, 281 µmol, **4**), potassium carbonate (21.6 mg, 618 µmol) and tetrakis(triphenylphosphine)-palladium(0) (21.6 mg, 18.7 µmol) were added and the mixture stirred for 46 h at 60 °C. The mixture was diluted with a saturated aqueous solution of sodium chloride (40 mL), deionized water (120 mL), and dichloromethane (120 mL). The layers were separated and the aqueous layer was extracted with dichloromethane (4 × 40 mL). The combined organic layers were dried over magnesium sulfate and the solvent was removed in vacuo. The crude product was absorbed on silica and purified by column chromatography (silica, toluene/diethyl ether/methanol + trifluoroacetic acid 45:45:10 + 0.1%, R_f = 0.3). The product was obtained as red solid. Yield: 199 mg (171 µmol, 91%)

M.p.: 250 °C, decomposition

^{1}H NMR (500 MHz, CD_2Cl_2, TFA-d_1, 298 K): δ = 8.91 (d, ^{3}J = 5.00 Hz, 2H, *H*-21), 8.81–8.77 (m, 4H, *H*-26/27), 8.67 (d, ^{3}J = 5.00 Hz, 2H, *H*-22), 8.31 (dd, ^{3}J = 7.46 Hz, ^{4}J = 1.08 Hz, 1H, *H*-15), 7.95 (td, ^{3}J = 7.80 Hz, ^{3}J = 1.31 Hz, 1H, *H*-17), 7.85 (td, ^{3}J = 7.50 Hz, ^{4}J = 1.31 Hz, 1H, *H*-16), 7.82 (dd, ^{3}J = 7.80 Hz, ^{4}J = 1.11 Hz, 1H, *H*-18), 7.72 (dd, ^{3}J = 7.55 Hz, ^{4}J = 1.40 Hz, 1H, *H*-6), 7.59 (d, ^{4}J = 1.29 Hz, 1H, *H*-2), 7.22 (ddd, ^{3}J = 7.80 Hz, ^{4}J = 1.56 Hz, ^{4}J = 1.02 Hz, 1H, *H*-12), 7.11 (ddd, ^{3}J = 7.84 Hz, ^{4}J = 1.90 Hz, ^{4}J = 1.10 Hz, 1H, *H*-10), 7.07 (t, ^{4}J = 1.80 Hz, 1H, *H*-8), 6.86 (t, ^{3}J = 7.85 Hz, 1H, *H*-11), 6.59 (d, ^{3}J = 7.50 Hz, 1H, *H*-5), 2.40 (s, br, 6H, CH_3) ppm.

^{13}C NMR (151 MHz, CD_2Cl_2, TFA-d_2, 298 K): δ = 154.5 (*C*-4), 151.3 (*C*-7), 144.9 (*C*-Porph.), 143.6 (*C*-Porph.), 143.6 (*C*-13), 143.3 (*C*-Porph.), 143.1 (*C*-9), 143.1 (*C*-Porph.), 138.4 (*C*-14), 137.2 (*C*-6), 136.4 (*C*-3), 136.3 (*C*-15), 134.9 (*C*-21), 134.3 (*C*-12), 132.6 (*C*-26/27), 132.4 (*C*-26/27), 131.7 (*C*-22), 130.3 (*C*-18), 130.1 (*C*-17), 129.4 (*C*-11), 128.6 (*C*-2), 127.2 (*C*-16), 123.2 (*C*-8), 123.0 (*C*-10), 121.1 (*C*-19), 110.6 (*C*-5), 30.2 (*C*-H3) ppm.

Signals of porphyrin carbon atoms, which could not be assigned to a specific position were marked as *C*-Porph.

^{19}F NMR (471 MHz, CD_2Cl_2, TFA-d_1, 298K): δ = −137.31 to −138.80 (m, 6F, *ortho-F*), −152.55 to −152.79 (m, 3F, *para-F*), −161.95 to −162.87 (m, 6F, *meta-F*) ppm.

MALDI-TOF-MS (Cl-CCA): *m*/*z* = 1165.1 $[M - H]^+$.

HR-ESI-MS (70 eV): *m*/*z* = 1165.13889 (calcd. for $C57H26F15N8Ni^{+}$), 1165.13862 (found) (−0.23 ppm).

FT-IR (ATR): ν = 2923 (m), 2853 (w), 1609 (m), 1517 (s), 1484 (s), 1373 (m), 1339 (m), 1290 (w), 1208 (w), 1158 (w), 1078 (m), 1053 (m), 985 (s), 955 (m), 937 (s), 926 (s), 838 (m), 799 (m), 761 (s), 743 (s), 705 (m), 696 (m), 658 (m), 621 (w), 585 (m), 501 (m), 425 (m) cm^{-1}.

UV–vis (*cis*, THF): $\lambda_{max.}$ (ε) = 428 (149610), 551 (8220) nm.

III: Photophysical experiments

Irradiation wavelengths

A solution of porphyrin **1** in tetrahydrofuran (11.2 µM) was irradiated with different wavelengths until the PSS was reached and the absorbance at 428 nm ($\lambda_{max.}$ paramagnetic *cis*-species) and 409 nm ($\lambda_{max.}$ diamagnetic *trans*-species) was determined via UV–vis spectroscopy (Figure S1).

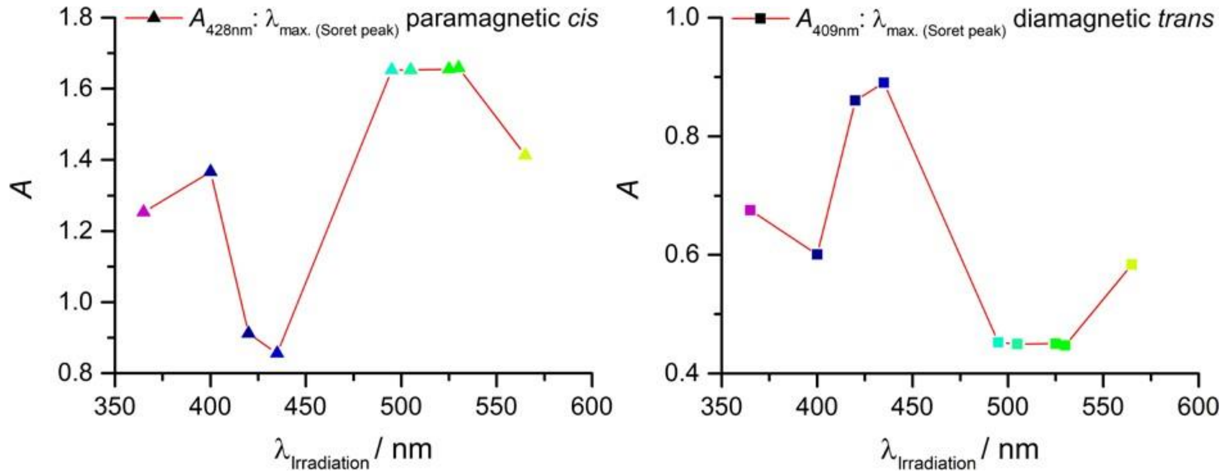

Figure S1: UV–vis absorbance of porphyrin **1** (11.2 µM in THF at 25 °C) in the PSS plotted as a function of the irradiation wavelength. Left (triangle data points): Absorbance at 428 nm ($\lambda_{max.}$ paramagnetic *cis* **1**). The highest amount of paramagnetic *cis* **1** was achieved, irradiating the sample with 530 nm. Right (square data points): Absorbance at

409 nm ($\lambda_{max.}$ diamagnetic *trans* **1**). The highest amount of diamagnetic *trans* **1** was achieved, irradiating the sample with 435 nm.

UV–vis experiments: switching efficiency of 1 in different solvents

30 µL of a stock solution of **1** in THF (601 µM) were added into a UV–vis cuvette and the solvent was slowly removed by compressed air. The cuvette was refilled with 3 mL of the corresponding solvent and the UV–vis spectra were recorded after irradiating the sample with 530 nm and 435 nm until the PSS was reached. THF, acetone, toluene, and dimethyl sulfoxide were investigated (Figure S2).

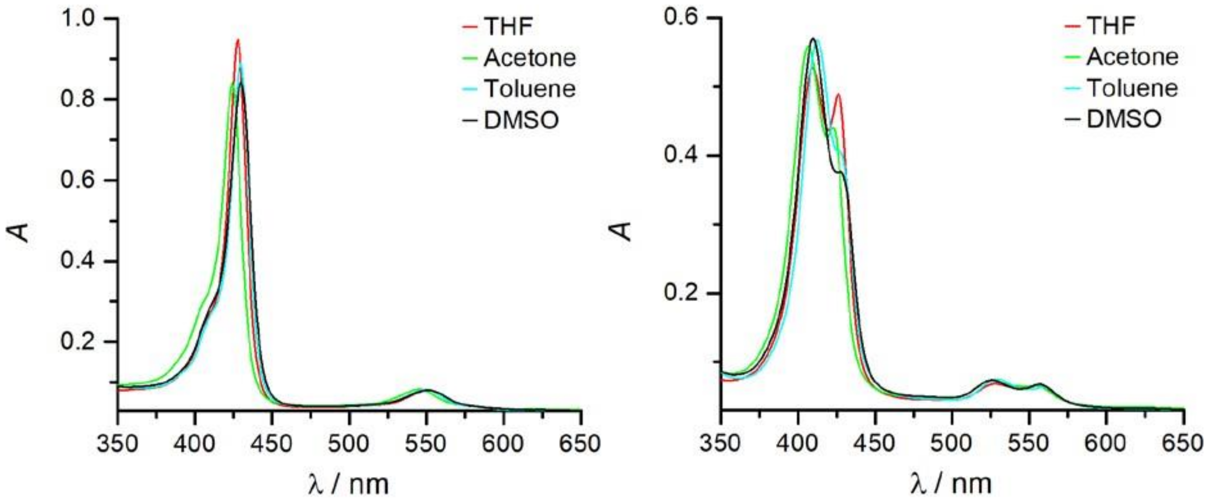

Figure S2: UV–vis spectra of porphyrin **1** (6.01 µM) in THF, acetone, toluene, and dimethyl sulfoxide after irradiation with 530 nm (left) and 435 nm (right) until the PSS was reached at 25 °C.

NMR experiments: switching efficiency of 1 in different solvents

1 mM stock solutions of porphyrin **1** in acetone-d_6, benzene-d_6, dimethyl sulfoxide-d_6, and tetrahydrofuran-d_8 were prepared and diluted to obtain a 1 mM and a 0.1 mM sample for NMR studies. Each sample was irradiated until the PSS was reached at 530 nm and 435 nm and the switching efficiency was investigated by ^{1}H NMR measurements (600 MHz). NMR investigations for record player molecules require to

differentiate between the *cis–trans* isomerization and the coordination/decoordination of the *cis*-isomer. The *cis–trans* ratio was analyzed by integration of *H*-11 because this signal suffers least from paramagnetic line broadening and overlap with other signals [2]. The results are given in Table S1.

Table S1: *Cis–trans* ratio in the PSS after irradiation with 530 nm and 435 nm in different solvents determined by ^{1}H NMR spectroscopy (600 MHz, 318 K).

Solvent	*c* [mM]	$\lambda_{Irradiation}$ [nm]	*cis*-peak [ppm]	*trans*-peak [ppm]	*cis*-ratio [%]	*trans*-ratio [%]
Acetone-d_6	1	530	6.71	6.82	70	30
		435			25	75
	0.1	530			75	25
		435			28	72
Benzene-d_6	1	530	6.42	6.15	51	49
		435			15	85
	0.1	530			56	44
		435			12	88
DMSO-d_6	1	530	6.85	6.78	62	38
		435			20	80
	0.1	530	No evaluation due to signal overlapping			
		435				
THF-d_8	4	530	6.58	6.48	93	7
		435			46	54
	1	530			96	4
		435			44	56
	0.1	530			95	5
		435			40	60

The ratio of coordinated ($\%_{cis\text{-coordinated}}$) vs. non-coordinated *cis*-isomer was determined by the chemical shift of the *cis*-pyrrole protons. The coordination/decoordination is fast on the NMR timescale. Thus, an average shift of coordinated and decoordinated *cis*-pyrrole protons was observed ($\delta_{obs.}$). The maximum downfield shift ($\delta_{max.}$) of the

pyrrole protons for a pure five-coordinated Ni-porphyrin was adopted from an intramolecular complex synthesized by Gutzeit *et al.* [3] The amount of coordinated *cis*-isomer is given in Table S2 and was calculated from the formula [3]:

$$\%_{cis-coordinated} = \frac{\delta_{obs.} - \delta_{dia.}}{\delta_{\max.} - \delta_{dia.}} \quad \text{(Equation S1)}$$

where:

$\%_{cis\text{-coordinated}}$	is the part of coordinated *cis*-**1** related to total *cis* amount.
$\delta_{obs.}$	is the observed average shift of the *cis*-**1** pyrrole protons in ppm.
$\delta_{dia.}$	is the shift of the total diamagnetic (ensured by TFA-d_1 addition) *trans*-**1** pyrrole protons in ppm.
$\delta_{max.}$	is the maximum shift (100 % coordinated) for a fivefold coordinated complex adopted from Gutzeit et al. [3] in ppm.

Table S2: Percentage of coordinated *cis* **1** determined by equation S1 and applied values for $\delta_{obs.}$, $\delta_{max.}$, and $\delta_{dia.}$ in different solvents. ^{1}H NMR spectra were recorded at 600 MHz.

Solvent	$\delta_{obs.}$ [ppm]	$\delta_{max.}$ [ppm]	$\delta_{dia.}$ [ppm]	Coordinated *cis*-**1** [%]
^{2}Acetone-d_6	48.40	48.86	9.10	98.8
^{2}Benzene-d_6	49.25	49.55	8.62	99.3
^{3}DMSO-d_6	49.98	49.98	10.56	100
^{3}THF-d_8	50.37	50.40	10.83	99.9

^{2}: 298 K ^{3}: 300 K

The composition of the different states (*cis*-coordinated, *cis*-decoordinated and *trans*) of porphyrin **1** in the PSS at 530 nm and 435 nm are shown in Figure 2 in the main text.

Switching efficiency of 1 at higher concentration

Porphyrin **1** (11.9 mg, 10.2 µmol) was dissolved in 255 µL THF-d_8, to obtain a 40 mM solution, which was transferred into a 3 mm NMR tube as used in the catalytic measurements. The sample was irradiated with 530 nm and afterwards with 435 nm under strict cooling and 1.50 µL of the sample were removed periodically (Table S3) and filled up with 600 µL THF-d_8 for NMR studies. ^{1}H NMR investigations were performed (600 MHz, 318 K) to analyze the irradiation time needed for the concentrated sample to reach the PSS and to determine the *cis*–*trans* ratio (Table S3).

Table S3: *Cis*-*trans* ratio of a 40 mM sample of **1** in THF-d_8 as used in the catalytic measurements after irradiation for different periods of time with light of 530 nm and 435 nm under strict cooling, determined by ^{1}H NMR spectroscopy (600 MHz, 318 K).

Irradiation wavelength [nm]	Irradiation time [h]	*cis*-ratio [%]	*trans*-ratio [%]
530	0.5	77	23
	1	83	17
	3	84	16
	9	83	17
435	0.5	40	60
	1	40	60
	3	40	60

Extinction coefficient

A stock solution of porphyrin **1** in tetrahydrofuran (601 µM) was diluted to obtain the concentrations shown in Figure S3 and irradiated with 530 nm until the PSS was reached. The absorbance at 428 nm and 551 nm was measured and plotted as a function of the concentration to calculate the extinction coefficient from the slope of the fitting line.

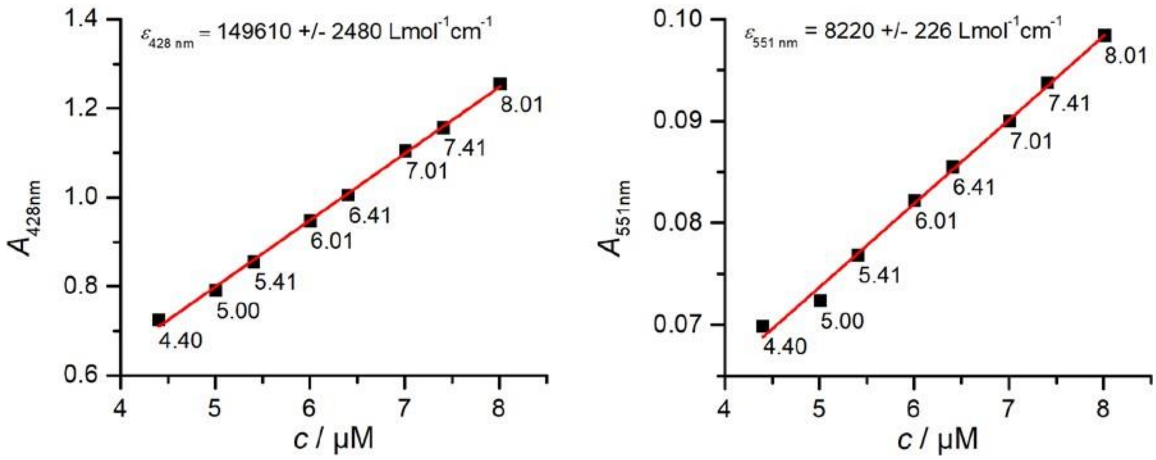

Figure S3: Plotting of the absorbance at 428 nm and 551 nm in UV–vis at 25 °C as a function of the concentration of porphyrin **1** in THF. The exact concentration for each measurement is given in µM at the data points.

Switching stability

A solution of porphyrin **1** in tetrahydrofuran (13.7 µM) was alternately irradiated with 530 nm and 435 nm into the PSS. UV–vis spectra were recorded after each irradiation cycle and the absorbance at 428 nm was measured (Figure S4). The data indicate a 20% loss in switching efficiency after 100 cycles.

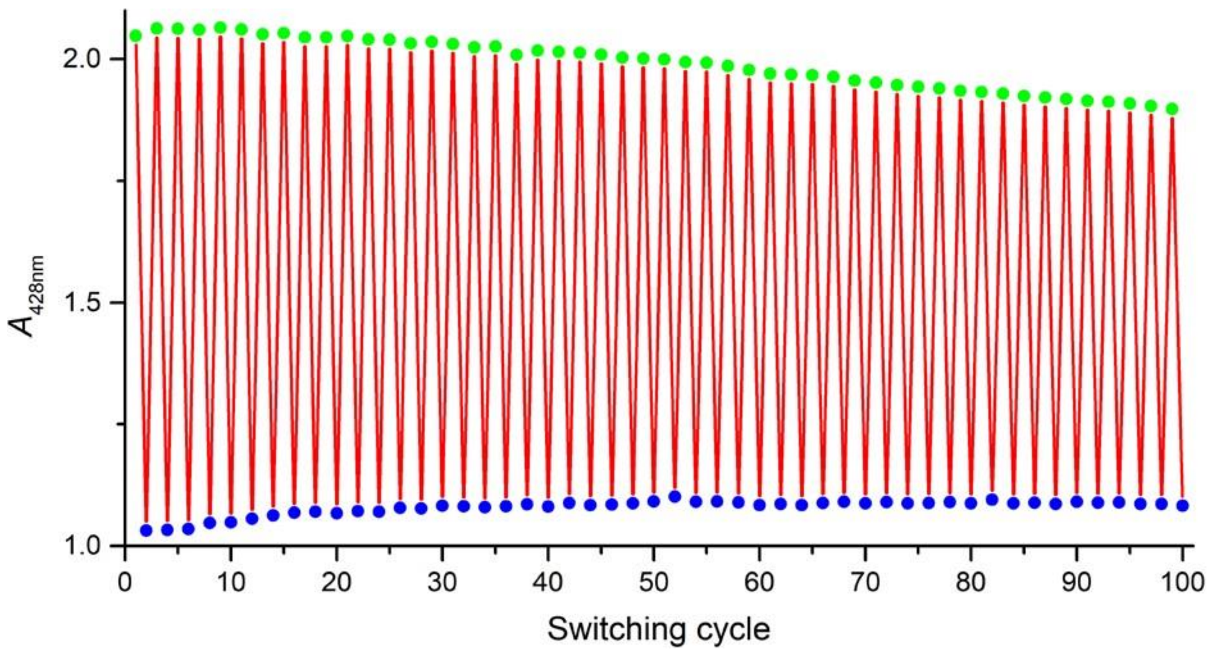

Figure S4: Absorbance at 428 nm of porphyrin **1** (13.7 µM in THF, 25 °C) after irradiation with 530 nm (green) and 435 nm (blue) plotted against the number of switching cycles.

Thermal half-life of *cis*-1

Porphyrin **1** (11.9 mg, 10.2 µmol) was dissolved in THF-d_8 (255 µL) to obtain a 40 mM stock solution, which was transferred into a 3 mm NMR tube. The sample was irradiated for 1 h to reach the PSS and stored at 25 °C (Huber Ministat 125 water bath). Periodically, 1.50 µL of the sample was removed and diluted with 600 µL THF-d_8. ^{1}H NMR spectra were recorded (600 MHz, THF-d_8, 318 K) and the *cis*/*trans* ratio was determined by integration of *H*-11 for the *cis* (6.58 ppm) and the *trans* (6.48 ppm) species (Figure S5). The thermal relaxation is a first order kinetic process, so *k* was determined by plotting ln(c/c_0) as a function of time (Figure S5) [4].

t [d]	*cis*-**1** [%]
0	83.53
7.01	82.22
27.89	79.62
37.88	79.39

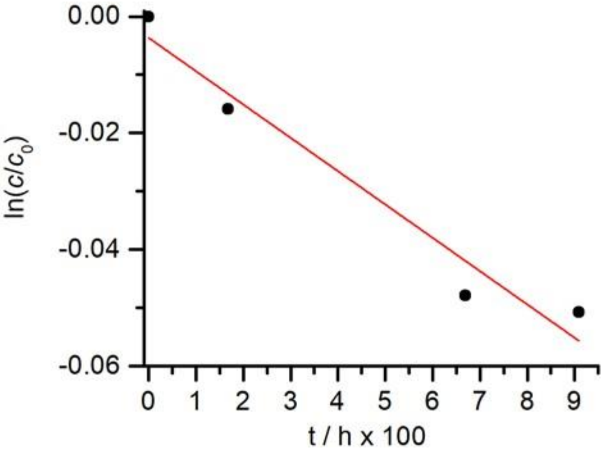

Figure S5: Determination of the thermal half-life of *cis*-**1** by measuring the decrease of the *H*-11 peak of the *cis* isomer **1** at 6.58 ppm (^{1}H NMR 600 MHz, in THF-d_8) as a function of time. After irradiation into the PSS at 530 nm the sample was kept at 25 °C and NMR spectra were measured in regular intervals (left). Plotting of ln(c/c_0) as a function of time revealed a first order rate constant of k = -5.72 × 10^{-5} (right).

The half-life period is given by formula [4]:

$$t_{1/2} = \frac{\ln(2)}{k} \qquad \text{(Equation S2)}$$

Due to the slow reaction and the long thermal half-life compared to the short period of measuring time, the calculated value of 505 days has an error bar of 89 days.

IV: Catalysis: Experimental procedure

Stock solutions of the compounds **6**, **8**, **2**, **4** and **1** in THF-d_8 (Table S4) were prepared and mixed with nitroethane (**5**) and THF-d_8 to obtain the sample compositions listed in Table S5.

Table S4: Stock solutions for the sample preparation.

c [mM]	Substance	*m* [mg]	*V* [mL]
814	4-Nitrobenzaldehyde (**6**)	615	5
34.0	Ni-TPPF$_{20}$ **8**	35.0	1
247	4-*N*,*N*-Dimethylaminopyridin (**2**)	75.3	2.5
120	*trans*-3-(3-Bromophenylazo)-4-*N*,*N*-dimethylaminopyridine (**4**)	36.6	1
244	*trans*-3-(3-Bromophenylazo)-4-*N*,*N*-dimethylaminopyridine (**4**)	34.2	0.46
24.1	4-N,N-Dimethylaminopyridine record player **1**	28.1	1

Table S5: Sample compositions for the catalytic measurements.

Sample	Catalyst	*c*(Catalyst) [mM] (mol%)	*cis/trans ratio*	*c*(**6**) [mM] (eq.)	*c*(**5**) [M] (eq.)
I	blank	0	-	400 (1)	4.78 (12)
II	**8**	4.02 (1)	-	400 (1)	4.80 (12)
III	**2**	4.10 (1)	-	400 (1)	4.79 (12)
IV	*trans*-**4**	3.99 (1)	0:100	400 (1)	4.79 (12)
V	**1** (PSS 530 nm)	4.02 (1)	93:7	400 (1)	4.79 (12)
VI	**1** (PSS 435 nm)	4.02 (1)	46:54	400 (1)	4.79 (12)
VII	**8**	40.2 (1)	-	400 (1)	4.80 (12)
VIII	**2**	40.0 (1)	-	400 (1)	4.86 (12)
IX	*trans*-**4**	40.2 (1)	0:100	400 (1)	4.80 (12)
X	**1** (PSS 530 nm)	40.2 (1)	83:17	400 (1)	4.80 (12)
XI	**1** (PSS 435 nm)	40.2 (1)	40:60	400 (1)	4.80 (12)

Due to solubility problems, sample VII, X, and XI were prepared by weighed amounts of the solid components. Towards this end 24.9 mg Ni-TPPF$_{20}$ **8** (24.1 µmol) was filled up with 206 µL nitroethane (**5**), 99 µL THF-d_8 and 295 µL of a 4- nitrobenzaldehyde (**6**) stock solution (814 mM) to obtain sample VII. For the preparation of sample X and XI 28.1 mg (24.1 µmol) 4-*N*,*N*-dimethylamino recordplayer **1** was filled up with 206 µL nitroethane (**5**), 99 µL THF-d_8 and 295 µL of a 4-nitrobenzaldehyde (**6**) stock solution (814 mM). The reaction mixture was divided and irradiated with 530 nm (sample X) and 435 nm (sample XI). ^{1}H NMR measurements (200 MHz, THF-d_8, 298 K) were performed. Signals at 8.50–8.30 ppm (starting material **6**, m, 2H, sample I–IX) or 10.08 ppm (starting material **6**, s, 1H, sample X,XI) and 7.80–7.60 ppm (product **7**, m, 2H) were analyzed to determine the conversion by integration (Figure S6 and Figure S7). The samples were stored at 25 °C (Huber Ministat 125 water bath) outside the spectrometer.

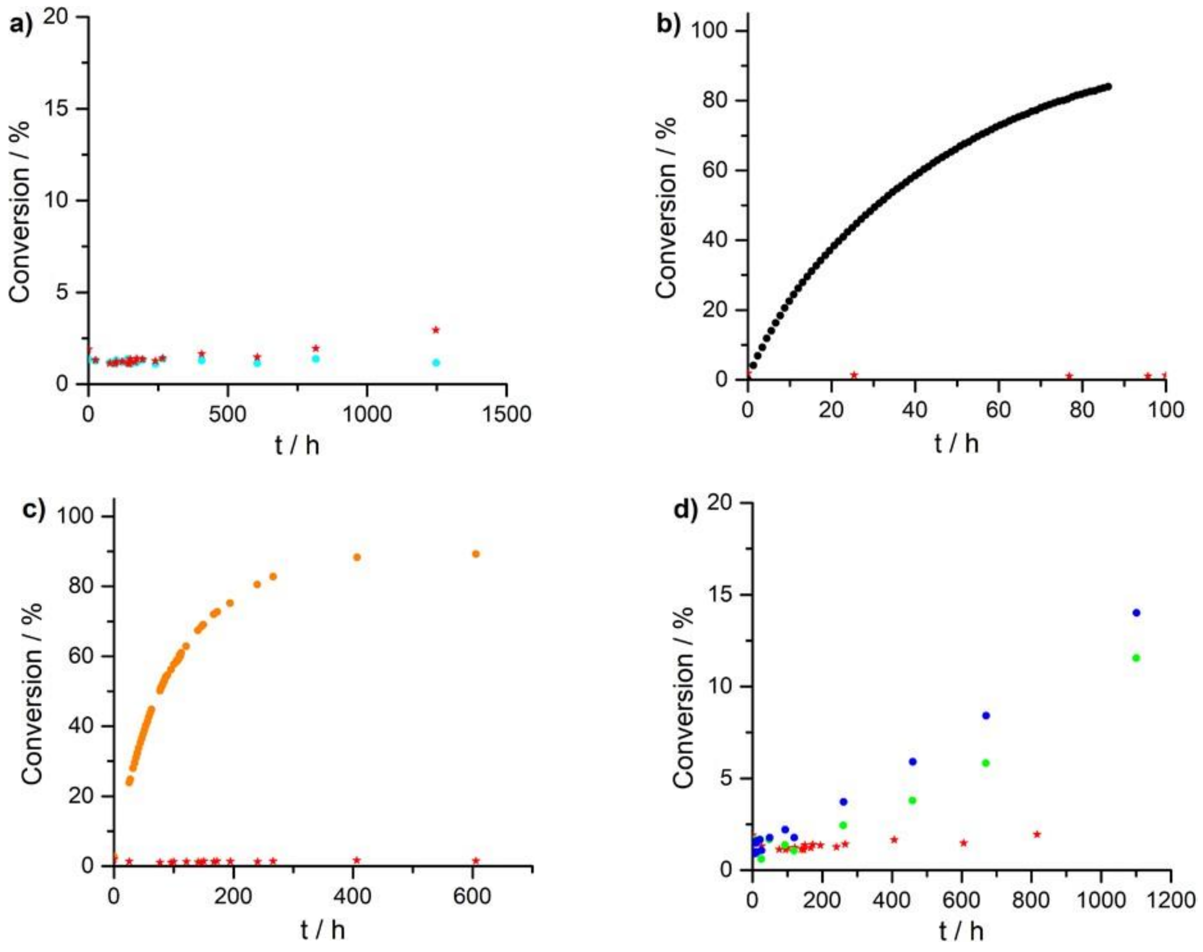

Figure S6: Conversion of 4-nitrobenzaldehyde (**6**) in the Henry reaction at 25 °C, determined by ^{1}H NMR spectroscopy (200 MHz, 298 K, THF-d_8), as a function of time using 1 mol % of **a)** Ni-TPPF$_{20}$ **8** (cyan dots), **b)** DMAP (**2**, black dots), **c)** NMe$_2$-azopyridine **4** (orange dots), **d)** *cis*-record player **1** (green dots), *trans*-record player **1** (blue dots) as catalyst. The conversion in the blank sample without catalyst is shown in each plot as red stars.

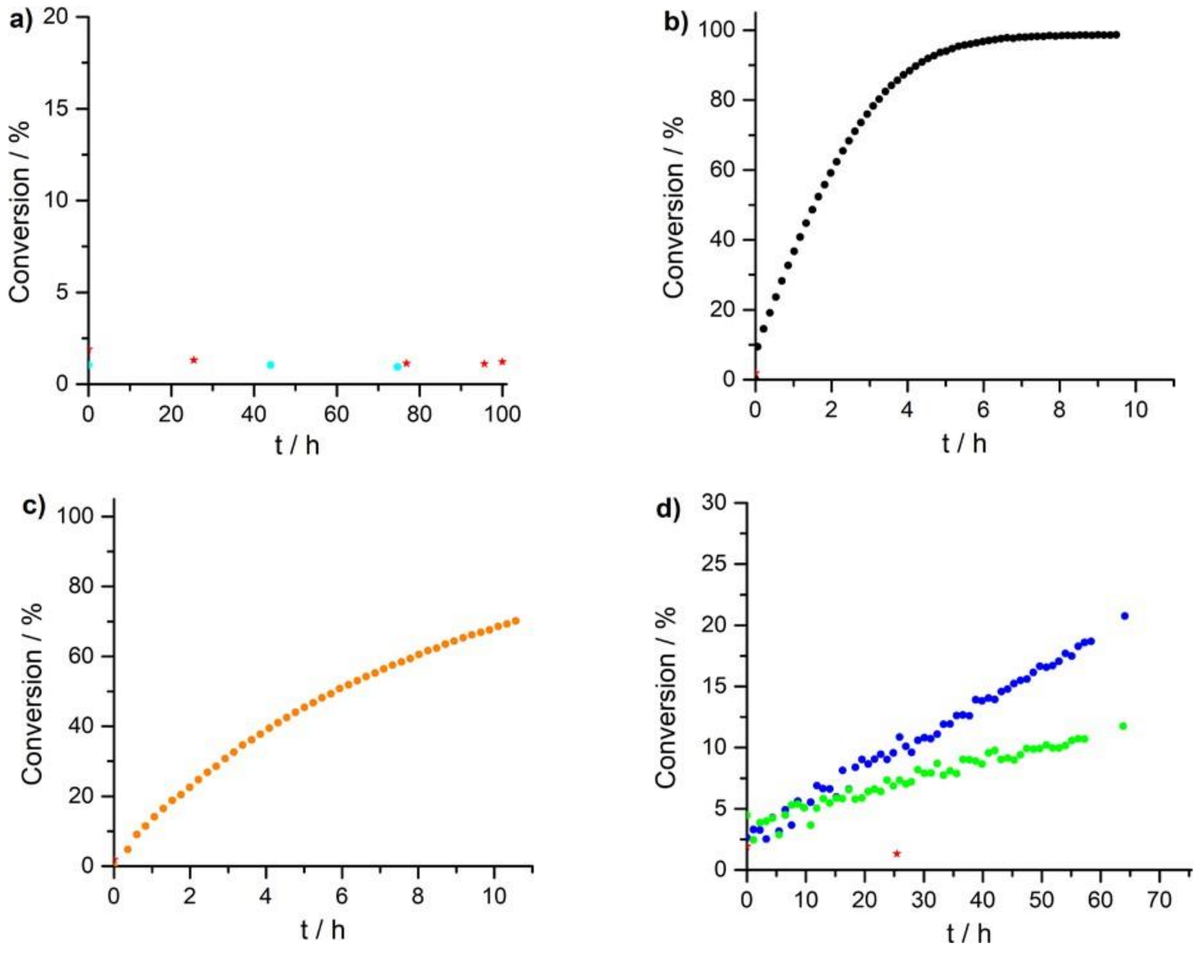

Figure S7: Conversion of 4-nitrobenzaldehyde (**6**) in the Henry reaction at 25 °C, determined by ^{1}H NMR spectroscopy (200 MHz, 298 K, THF-d_8), as a function of time using 10 mol % of **a)** Ni-TPPF$_{20}$ **8** (cyan dots), **b)** DMAP (**2**, black dots), **c)** NMe$_2$-azopyridine **4** (orange dots), **d)** *cis*-record player **1** (green dots), *trans*-record player **1** (blue dots) as catalyst. The conversion in the blank sample without catalyst is shown in each plot as red stars.

Due to the large excess of nitroethane **5** we assume pseudo first order kinetics and a rate determining deprotonation of nitroethane. The rate law is given by formula [4]:

$$\frac{d[6]}{dt} = -k_{tot.}[6] \qquad \text{(Equation S3)}$$

Plotting ln(c/c_0) of 4-nitrobenzaldehyde (**6**) as a function of the reaction time and linear regression provides the rate constants of the total reaction ($k_{tot.}$) obtained as the negative slope for the different samples (Figure S8). Note that no or a very slow conversion was observed in the relevant reference period in the presence of Ni-TPPF$_{20}$ **8** (Figure S8 II, S6a/S7a).

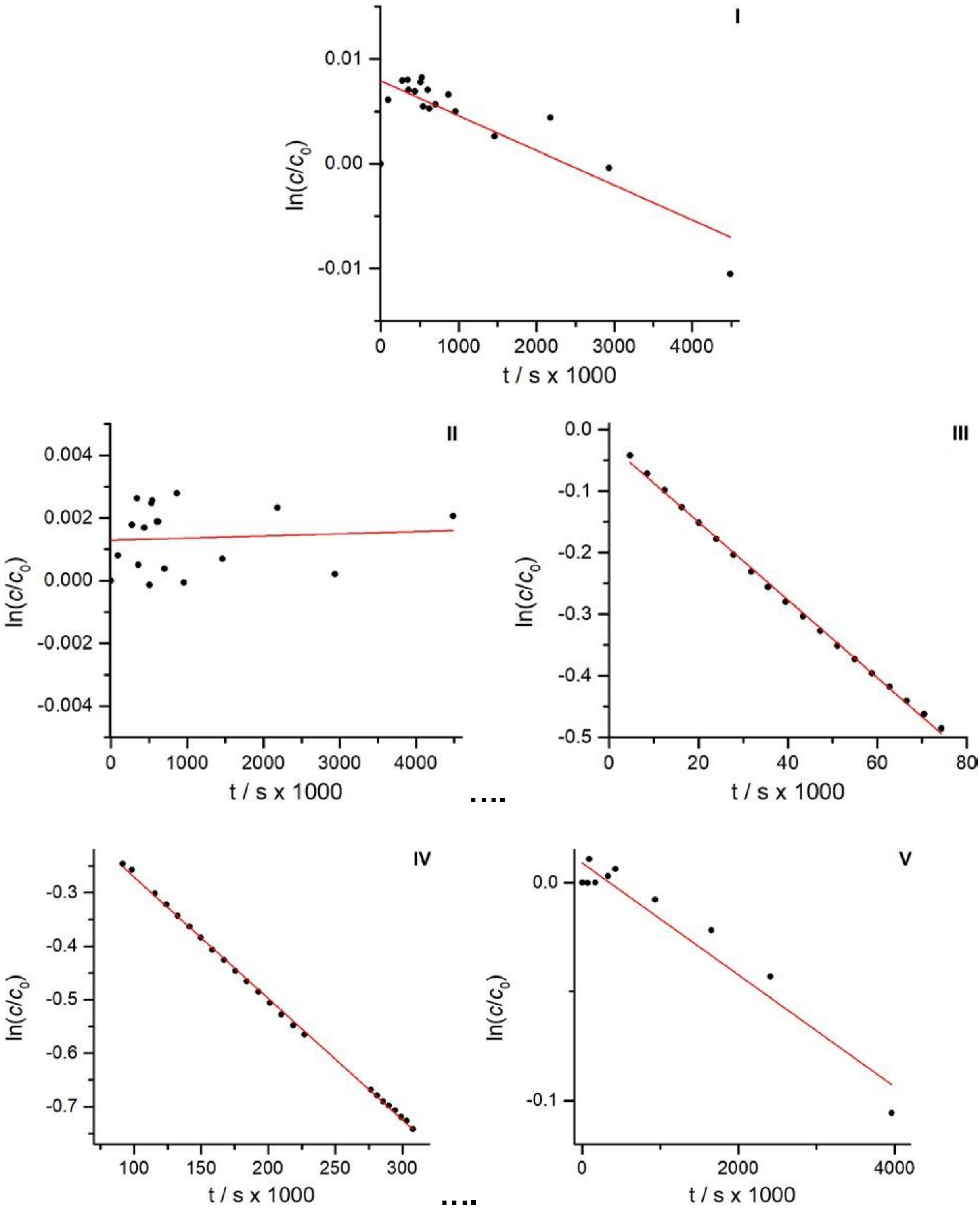

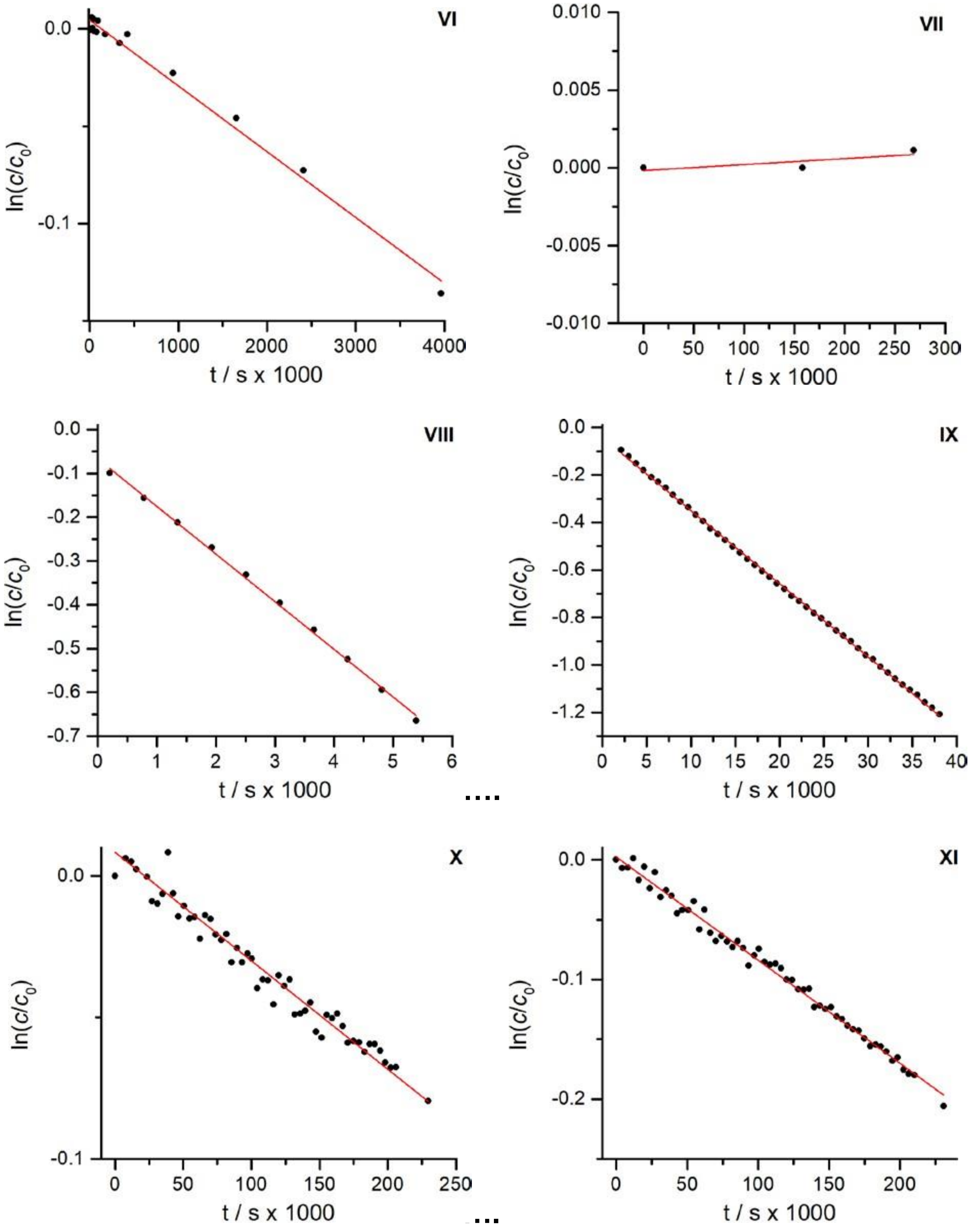

Figure S8: Plots of ln(c/c_0) of 4-nitrobenzaldehyde (**6**) (determined by ^{1}H NMR spectroscopy, 200 MHz, 298 K, THF-d_8) as a function of the reaction time. The rate constants of the total reaction ($k_{tot.}$) were obtained by linear regression as the negative slope for the different samples (for sample numbering see Table 1).

Calculation of the rate constant k_0, assuming the presence of pure *trans*-1 and neglecting any intermolecular coordination

Two major factors reduce the catalytic switching efficiency: 1. Incomplete photochemical conversion to the active *trans* isomer of **1** and incomplete conversion to the inactive *cis* form. 2. Intermolecular coordination, which reduces the catalytic efficiency of the *trans* isomer. To estimate the extend of these limiting factors, we assumed perfect reaction conditions, namely, 100% photochemical conversion in both directions and no intermolecular coordination. Towards this end, the amount of uncoordinated *trans*-**1** was calculated according to equation S4 and multiplied with the amount of isomer in the considered PSS obtained from Table S1/S3 (Table S6).

$$\%_{uncoordinated-trans} = 1 - \frac{\delta_{obs.} - \delta_{dia.}}{\delta_{\max.} - \delta_{dia.}} \qquad \text{(Equation S4)}$$

where:

$\%_{unoordinated\text{-}trans}$	is the part of uncoordinated *trans*-**1** related to total *trans* amount.
$\delta_{obs.}$	is the observed average shift of the *trans*-**1** pyrrole protons in ppm.
$\delta_{dia.}$	is the shift of the total diamagnetic (ensured by TFA-d_1 addition) *trans*-**1** pyrrole protons in ppm.
$\delta_{max.}$	is the maximum shift observed for a completely coordinated Ni complex in ppm.

Table S6: Calculated catalytically active percentage of *trans*-**1** in the PSS at 530 nm and 435 nm (4 mM and 40 mM) and the corresponding rate constants ($k_{tot.}$, Table 6).

c(**1**) [mM]	PSS	catalytically active *trans*-**1** [%]	$k_{tot.}$ [s^{-1}].
4	530 nm	6.5	2.57×10^{-8}
4	435 nm	50.2	3.38×10^{-8}

40	530 nm	12.7	3.84×10^{-7}
40	435 nm	45.0	8.62×10^{-7}

The obtained value of catalytically active *trans*-**1** was plotted as a function of the determined rate constant ($k_{tot.}$). Linear fitting (Figure S9) provided the rate constants for remaining catalytic activity of *cis*-**1** (catalytically active *trans*-**1** = 0%) and completely catalytically active *trans*-**1** (catalytically active *trans*-**1** = 100%).

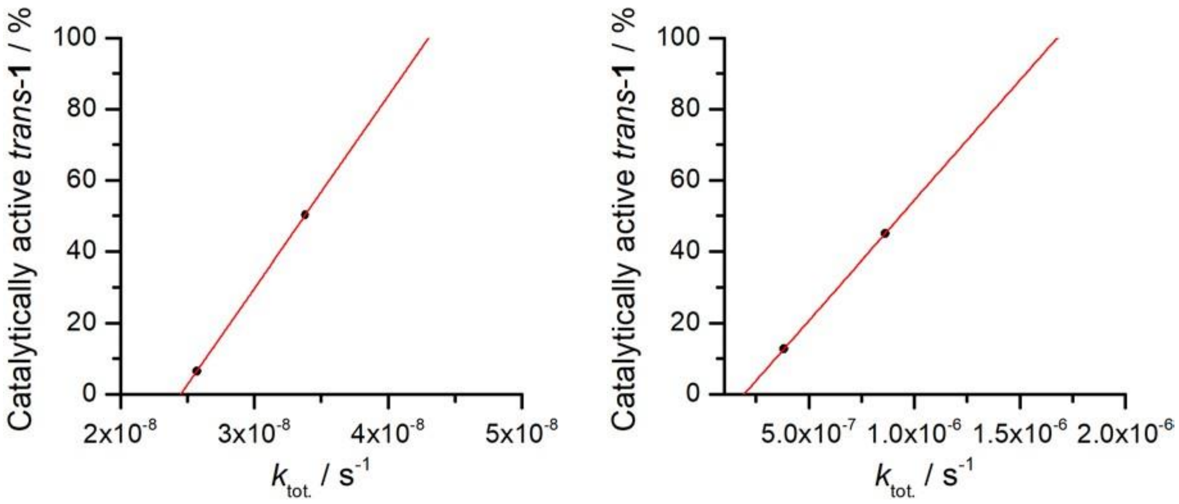

Figure S9: Catalytically active percentage of *trans*-**1**, plotted as a function of the rate constant ($k_{tot.}$) for 4 mM (left) and 40 mM (right). A linear fitting was assumed to calculate k_0 for 100 und 0% catalytically active *trans*-**1** (Table 1 and Table S7).

Table S7: Hypothetical rate constants k_0 assuming 100% photoconversion in both directions and neglecting intermolecular coordination.

catalyst (mol %)	K_0
cis- NMe_2-record player **1** (1%)	2.45×10^{-8}
trans- NMe_2-record player **1** (1%)	4.30×10^{-8}
cis- NMe_2-record player **1** (10%)	1.95×10^{-7}
trans- NMe_2-record player **1** (10%)	1.68×10^{-6}

V: NMR spectra

NMR spectra of 5-(biphenylazo-*N,N*-dimethylaminopyridine)-10,15,20-tris(pentafluorophenyl)nickel(II)porphyrin (1)

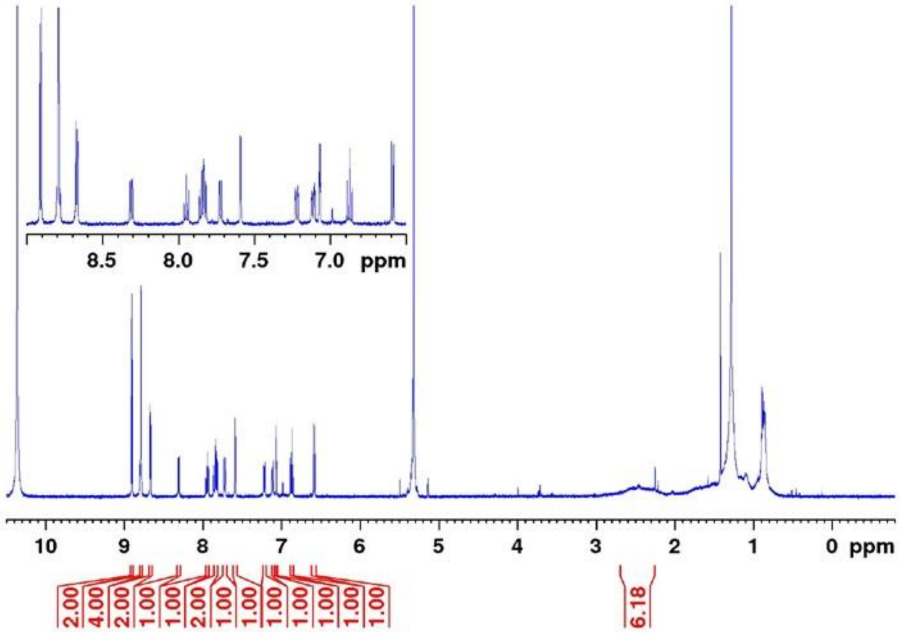

Figure S10: ^{1}H NMR spectrum of **1** with enlarged section of the aromatic area.

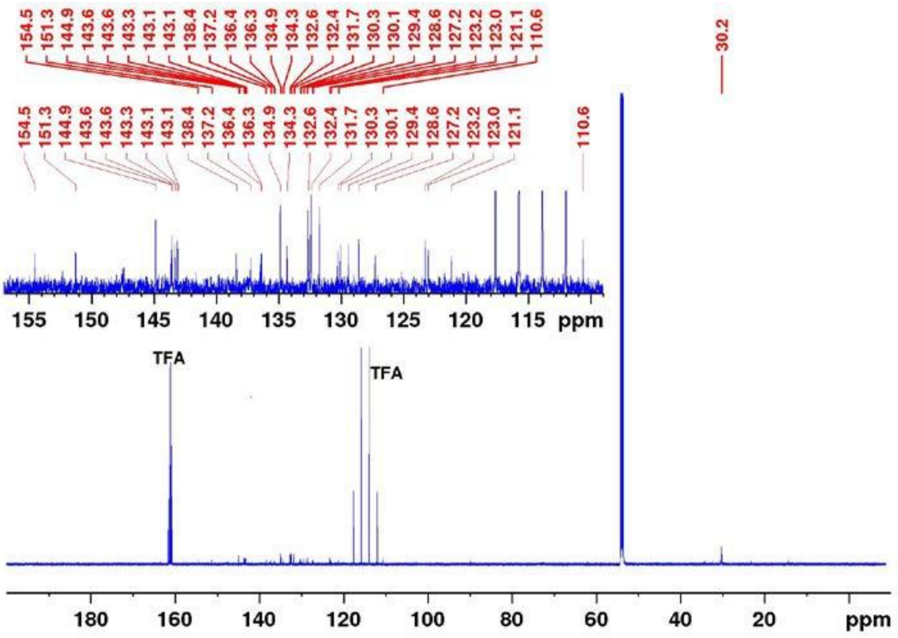

Figure S11: ^{13}C NMR spectrum of **1** with enlarged section of the aromatic area.

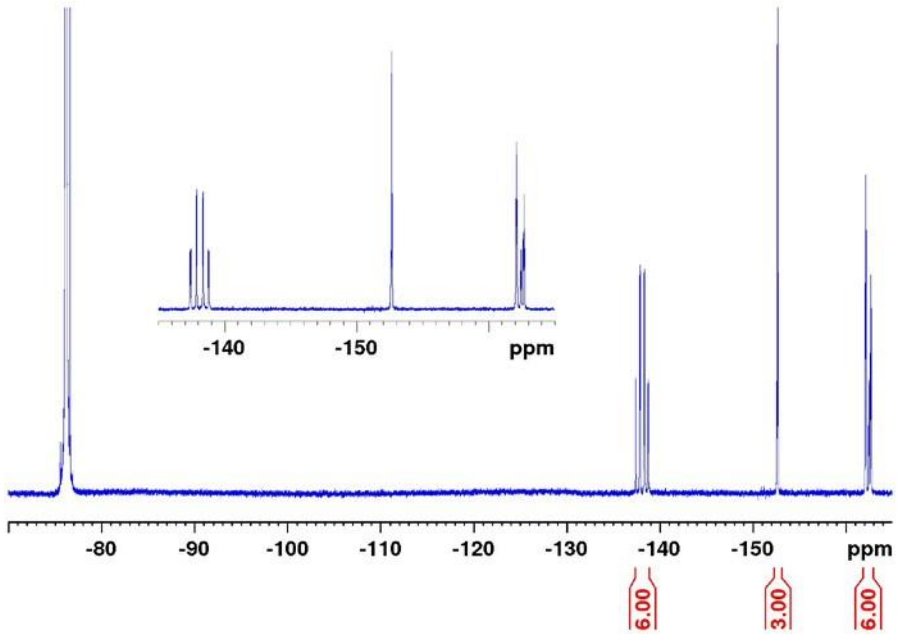

Figure S12: ^{19}F NMR spectrum of **1** with enlarged section of the relevant area.

VI: References

1. Fulmer, G. R.; Miller, A. J. M.; Sherden, N. H.; Gottlieb, H. E.; Nudelman, A.; Stoltz, B. M.; Bercaw, J. E.; Goldberg, K. I. *Organometallics,* **2010,** *29,* 2176–2179.

2. Dommaschk, M.; Peters, M.; Gutzeit, F.; Schütt, C.; Näther, C.; Sönnichsen, F. D.; Tiwari, S.; Riedel, C.; Boretius, S.; Herges, R. *J. Am. Chem. Soc.,* **2015**, *137,* 7552–7555.

3. Gutzeit, F.; Dommaschk, M.; Levin, N.; Buchholz, A.; Schaub, E.; Plass, W.; Näther, C.; Herges, R. *Inorg. Chem.*, **2019**, 12542–12546.

4. Atkins, P.; de Paula, J. 22 The Rates of Chemical Reactions. In *Atkins´ Physical Chemistry*; Oxford University Press: Oxford 2006; Vol. 8, p 800-810.
